# Isolation, identification, recombination analysis and pathogenicity experiment of a PRRSV recombinant strain in Sichuan Province, China

**DOI:** 10.3389/fmicb.2024.1362471

**Published:** 2024-02-21

**Authors:** Teng Tu, Yanwei Li, Guidong Zhang, Chengchao Du, You Zhou, Dike Jiang, Yan Luo, Xueping Yao, Zexiao Yang, Meishen Ren, Yin Wang

**Affiliations:** ^1^Key Laboratory of Animal Disease and Human Health of Sichuan Province, Sichuan Agricultural University, Chengdu, China; ^2^College of Veterinary Medicine, Sichuan Agricultural University, Chengdu, China

**Keywords:** porcine reproductive and respiratory syndrome virus (PRRSV), genetic recombination, lineage 1.8, pathogenicity, preventing and controlling

## Abstract

Since 2013, the porcine reproductive and respiratory syndrome virus type 2 (PRRSV-2), lineage 1.8 (NADC30-like PRRSV) has emerged and become widely prevalent in China. The NADC30-like PRRSV poses significant challenges for disease control, primarily because of its propensity for frequent mutations and recombinations. We successfully isolated and identified a NADC30-like strain, designated SCCD22, in Chengdu, Sichuan Province, China. We meticulously examined the genetic recombination properties and evaluated its pathogenicity in 28-day-old piglets. SCCD22 showed 93.02% nucleotide homology with the NADC30 PRRSV strain, and its non-structural protein 2 coding region showed the same 131 amino acid deletion pattern as that seen in NADC30. Furthermore, we identified two recombination events in SCCD22: one in the NSP2 region (1,028–3,290 nt), where it was highly similar to the JXA1-like strain GZ106; and another in the NSP10 ~ 12 region (9,985–12,279 nt), closely resembling the NADC30-like strain CY2-1604. Piglets infected with SCCD22 exhibited clinical symptoms such as elevated body temperature, prolonged fever, reduced appetite, and roughened fur. Postmortem examinations underscored the typical lung pathology associated with PRRSV, indicating that the lungs were the primary affected organs. Furthermore, extended viral shedding accompanied by progressive viremia was observed in the serum and nasal excretions of infected piglets. In summary, this study reports a domestic PRRSV recombination strain in the Sichuan Province that can provide critical insights into preventing and controlling PRRSV in this region.

## 1 Introduction

Porcine reproductive and respiratory syndrome (PRRS) is a highly contagious disease caused by the porcine reproductive and respiratory syndrome virus (PRRSV). It differs from clinical manifestations caused by other pathogens such as African swine fever virus, classical swine fever virus, porcine parvo virus, and porcine circovirus type 2, and it leads to severe reproductive disorders, delayed growth, respiratory symptoms, and high mortality rates in pigs (Kappes and Faaberg, 2015).

PRRSV is a single-stranded positive-sense RNA virus that belongs to the Arteriviridae family. The genome size of PRRSV ranges from 15 to 15.5 Kb, encoding approximately 10 open reading frames (ORFs), which include ORF1a, ORF1b, ORF2a, ORF2b, ORF3, ORF4, ORF5a, ORF5, ORF6, and ORF7. ORF1a and ORF1b together account for approximately 75% of the genome and encode at least 16 non-structural proteins (NSPs), such as NSP1a, NSPSP1β, NSP2, NSP2TF, NSP2N, NSP3-NSP6, NSP7α, NSP7β, and NSP8-NSP12 (Jiang et al., 2023). ORF2a, ORF2b, ORF3, ORF4, ORF5, ORF6, and ORF7 encode seven structural proteins: glycoprotein GP2a, small envelope protein E, glycoproteins GP3, GP4, and GP5, membrane protein (M protein), and nucleocapsid protein (N protein) (Meng et al., 1995; Kappes and Faaberg, 2015; Lunney et al., 2016).

Based on differences in genomic sequences and antigenic characteristics, the prevalence of PRRSV can be divided into PRRSV-1 (represented by the European type with the lelystadvirus strain) and PRRSV-2 (represented by the North American type with the VR-2332 strain) (Adams et al., 2016). Shi constructed a global classification system for PRRSV based on a comprehensive analysis of the complete ORF5 gene sequence (Shi et al., 2010b). According to this classification system, PRRSV-1 can be divided into three subtypes (sublineages 1–3). Although numerous reports have been published on the prevalence of PRRSV-1 in China in recent years, its clinical detection rate remains relatively low. As early as 1997, Chinese customs intercepted pigs infected with PRRSV-1 (B13, GenBank: AY633973) (Yun et al., 1998), indicating that the introduction of PRRSV-1 may have occurred more than 20 years ago.

PRRSV-2 is divided into nine lineages, each with several sub-lineages (Shi et al., 2010a,b). The virulence and antigenicity of each lineage vary owing to genetic diversity. Five lineages of PRRSV-2 have been reported in China, namely lineages 1 (sublineages 1.5 and 1.8), 3, 5 (sublineage 5.1), 8 (sublineage 8.7), and 9 (Chen et al., 2019; Han et al., 2020). Lineage 1 of PRRSV includes representative strains such as NADC30, JL580, NADC34, and RFLP1-4-4; lineage 3 is mainly prevalent in southern China, with relatively low pathogenicity, including representative strains QYYZ and GM; lineage 5 mainly contains the classic PRRSV strain represented by VR-2332; lineage 8 contains highly pathogenic strains represented by TJ, JXA1, TA-12, and the classic strain represented by CH-1a; lineage 9 was discovered in Xinjiang in 2011.

Sublineage 1.8 (NADC30-like) of PRRSV-2 has been reported in China since 2013 (Zhou F. et al., 2015). Similar to the American NADC30 isolates MN184A and NADC30, the Chinese NADC30-like virus strains exhibit the same NSP2 deletion pattern, including a discontinuous deletion of 131 amino acids (Feng et al., 2014; Zhao et al., 2015; Zhou L. et al., 2015; Li et al., 2016). Recently, multiple novel recombinant PRRSV strains from NADC30-like outbreaks in China have recently been reported to exhibit different pathogenicities (Zhao et al., 2015; Liu et al., 2017; Chen et al., 2018). Compared with other PRRSV lineages in China, the NADC30-like strain has a higher potential for recombination and pathogenic diversity. Numerous recent studies (Zhao et al., 2015; Zhang et al., 2016; Liu et al., 2018; Sui et al., 2018; Han et al., 2019; Zhang et al., 2019; Li et al., 2021; Liu et al., 2022; Ma et al., 2022; Wu et al., 2022; Chang et al., 2023) have indicated that the virulence of NADC30-like PRRSV strains is related to the recombination regions and segments derived from the parental strains. Currently, all NADC30-like PRRSV strains induce typical clinical symptoms and pathological changes in piglets following infection, with their pathogenicity tending toward intermediacy between the parental strains (Yu et al., 2021).

This study aims to elucidate the genetic and pathogenic features of PRRSV in Sichuan Province, through the isolation, recombination analysis and pathogenicity experiment of a NADC30-like PRRSV strain. In this study, the Pams-163 (PRRSV receptor CD163 was expressed and transfected into primary alveolar macrophage passage cell line 3D4/21 (CRL-2843). The Pams-163 was maintained and provided by the Animal Quarantine Laboratory of Sichuan Agricultural University) was used to isolate and identify prevalent PRRSV strains from suspected PRRSV-infected samples, resulting in the isolation of an NADC30-like PRRSV strain. The isolated strain underwent whole-genome sequencing, genetic evolution analysis, and pathogenicity testing in piglets to clarify the genetic evolutionary relationship and recombination events of the isolated strain and to assess its pathogenicity in 28-day-old piglets. The results of this study lay the foundational scientific groundwork for future prevention and control strategies against PRRSV in Sichuan Province. Firstly, our comprehensive genome-wide analysis of the isolated strains holds promise for identifying novel genetic markers. These markers could be instrumental in developing specific vaccines and diagnostic assays, meticulously tailored to combat potential NADC30 PRRSV outbreaks. Secondly, a deeper understanding of the unique mutation and recombination patterns of this virus could inform the creation of more targeted antiviral therapeutics. Furthermore, the pathogenicity assessment conducted in this study offers valuable insights into the virulence of this newly identified PRRSV variant, which is crucial for formulating effective disease management strategies.

## 2 Materials and methods

### 2.1 Sample collection and processing

In 2022, samples suspected of PRRSV infection, including serum, lung tissue, saliva swabs, semen, were collected from a pig farm in Chengdu, Sichuan Province, China. After subjecting the samples to three freeze–thaw cycles, where each cycle involved freezing at −80°C for 15 min followed by thawing at room temperature (26°C), 2 mL of PBS was added to the samples. They were then centrifuged at 12,000 rpm for 5 min at 4°C. Following centrifugation, the samples were stored in an ultra-low temperature freezer at −80°C for subsequent pathogen detection and virus isolation.

### 2.2 RNA extraction and reverse transcription

Total RNA was extracted from the samples using the AG RNAex Pro RNA extraction reagent (Accurate Biology Co., Ltd., Hunan, China), following the manufacturer’s instructions. Subsequently, cDNA was synthesized using the Evo M-MLV RT Kit (Accurate Biology Co., Ltd., Hunan, China) according to the manufacturer’s protocol. The reverse transcription reaction system and conditions are listed in Supplementary Table S1.

### 2.3 Primer design and PRRSV detection

The cDNA obtained from 2.2 was detected using RT-qPCR to confirm PRRSV positive. All primers used are listed in Table 1. All primers were synthesized by TsingkeBiotechnology Co., Ltd. (Chengdu, China).

**Table 1:** Information for detection primer.

Type of virus	Sequence (5′-3′)	Target gene	Length of products (bp)	References
NADC34-like PRRSV	F: CCTGTGTGACTCATATTGTCTCC R: CGGCGTAAATGCTACTCAAGAC P: FAM-CGCCCTCACCACCAGCCATTTCCT-BHQ1	ORF5	129	Tu et al. (2023)
HP-PRRSV	F: GACGTGCCCCCAAGCTGAT R: GGATGCCCATGTTCTGCGA P: FAM-CGTAGAACTGTGACAACAACGCTGAC-BHQ1	*NSP2*	171	Qiu et al. (2020)
NADC30-likePRRSV	F: CGTATTGGACACCTCTTTTGACTG R: AACTGGACCTAATCTTCCTGCG P: ROX-CCCAAAGGTCTTCGTCGGTATTCC-BHQ2	*NSP2*	218	Qiu et al. (2020)
Classical PRRSV	F: GCAATTGTGTCTGTCGTC R: CTTATCCTCCCTGAATCTGAC	ORF5	81	Chai (2009)

### 2.4 Isolation and identification of PRRSV

To increase the volume of viral fluid for the isolation of PRRSV, we meticulously selected approximately 5 g of lung tissue that was confirmed to be PRRSV-positive using RT-qPCR. This tissue was finely minced using scissors and ground in a mortar. After adding PBS and undergoing three freeze–thaw cycles, the supernatant was collected and sterilized using a 0.22 μm filter, then stored at −80°C for later use. A 500 μL aliquot of the treated supernatant was added to a monolayer of Pams-163 (constructed, maintained and provided by the Animal Quarantine Laboratory of Sichuan Agricultural University) with a confluence of approximately 90%. After a one-hour incubation period, the supernatant was meticulously removed. The cells were subsequently incubated for 48 h in a 5% CO_2_ incubator at 37°C. Following this incubation, PRRSV was detected using a fluorescein isothiocyanate-labeled polyclonal antibody specifically targeting the PRRSV N protein (Bioss, Beijing, China).

In the subsequent phase, the purified viral fluid was subjected to a 10-fold serial dilution and inoculated onto a 96-well cell culture plate. Following a 5-day incubation at 37°C in a CO_2_-controlled environment, the cytopathic effect (CPE) induced by PRRSV was meticulously evaluated using an inverted microscope (Olympus Corporation, Tokyo, Japan). The CPE was characterized by notable changes in Pams-163 cells, including cell shrinkage, detachment, and the blurring of cellular boundaries. The 50% tissue culture infectious dose (TCID_50_) of PRRSV was calculated using the Reed-Muench formula based on the number of wells with CPE.

Purified viral isolates were used to sequence their entire genomes. To investigate the effect of multiplicity of infection (MOI) on the growth of PRRSV in Pams-163, we infected cells with MOI = 0.01 in triplicate. We incubated them for 1.5 h at 37°C. RPMI 1640 medium containing 2% serum was added for maintenance, and virus fluid was collected every 24 h and frozen at −80°C. After 144 h, the CPE induced by PRRSV was assessed. Subsequently, the TCID_50_ of PRRSV was determined using the Reed-Muench formula. A viral growth curve was constructed based on the titration results. The PRRSV strain isolated from Sichuan Province was designated as SCCD22.

### 2.5 Whole-genome sequencing and genetic evolution analysis of SCCD22

PRRSV RNA was extracted, and cDNA was prepared according to the above procedures. Subsequently, RT-qPCR was conducted to verify that the tested sample contained an adequate number of viral copies, thus ensuring its suitability for further analysis (the qPCR cycle threshold value was <25). Once confirmed, the sample was sent to Beijing Tsingke Biotech Company (Beijing, China) for whole-genome sequencing. Illumina sequencing technology was used with a coverage of 5,000, and the assembly method used was FastPlast v1.2.6. The complete genome sequence of SCCD22 was annotated and visualized using CGView.^1^ Phylogenetic tree was constructed using the distance-based neighbor-joining method with 1,000 bootstrap replicates in MEGAX software (Tempe, AZ, United States) for the purpose of genetic evolutionary analysis.

### 2.6 Genetic recombination analysis of SCCD22

The SCCD22 strain was compared with the full-genome sequences of representative strains from various lineages in Genbank for recombination analysis using RDP5.1 and SimPlot 3.5.1. Recombination breakpoints in the sequences were predicted using RDPv5.1. A recombination event was confirmed if it was supported by at least three of the following methods: RDP, GENECONV, MAXCHI, CHIMAERA, Bootscan, Siscan, and 3SEQ. Similarity comparisons were further performed using SimPlot 3.5.1 within a 200 bp sliding window along the genomic alignment (20-bp step), and the complete SCCD22 genome was selected as the query sequence, and the reference sequences were strains GZ106, ISU18, NADC 30, and CY2-1604.

### 2.7 Pathogenicity analysis of SCCD22

To strike a balance between statistical significance and practical limitations, we opted for a sample size of 10 piglets for the pathogenicity experiment. Ten weaned piglets (five males and five females, breed Yorkshire, approximately 4 weeks old), were purchased from Chengdu Wangjiang Agriculture and Animal Husbandry Technology Co., Ltd., and the weight variance among all piglets in the study was within a range of 500 g. After testing negative for both PRRSV antibodies and antigens, the piglets were randomly divided into two groups and housed in separate rooms. Their breed, number and grouping in the experiment with the 3R principle. All animals were housed under controlled conditions with a temperature of 26°C, humidity of 60%, and 12 h of light per day, with *ad libitum* access to water. Group 1 (infection group): five piglets received intranasal inoculation with virus solution (TCID_50_ = 10^5^/mL), 2 mL per piglet. Group 2 (control group): five piglets received an intranasal inoculation of 2 mL of RPMI 1640 medium per piglet.

Following the virus inoculation, daily recordings were made of the rectal temperatures of the pigs, and their clinical manifestations were observed, such as appetite, mental state (presence of unconsciousness/coma), and respiratory condition. Clinical symptom scoring (Jiang et al., 2023) is detailed in Supplementary Table S2. Additionally, on days 3, 7, 10, and 14 post-inoculation, the pigs were weighed to calculate the average daily weight gain. These comprehensive assessments aimed to evaluate the impact of the viral infection on the health and welfare of the piglets and to provide insights into potential treatment strategies for this specific strain.

Blood samples were drawn from the anterior vena cava of the piglets using a 5 mL syringe at 0, 3, 5, 7, 10, and 14 days post-infection. Concurrently, nasal secretions were collected using cotton swabs. Subsequently, the shedding of the virus was detected using RT-qPCR. On days 7, 10, and 14, viremia was measured using the TCID_50_ method. This study aimed to gain a deeper understanding of the viral shedding and clearance mechanisms in piglets, thereby enhancing our understanding of how this specific virus strain interacts with its host.

Throughout the experiment, the survival of the piglets in each group was monitored. On day 14 post-infection, all piglets were humanely euthanized. Necropsies were immediately performed on the dead piglets to observe gross pathological changes in various organs. Lung tissues were collected and fixed in a 4% paraformaldehyde solution. After fixation, tissue embedding, paraffin sectioning, and hematoxylin and eosin (H&E) staining were performed for pathological examination. Additionally, the spleen, lungs, thymus, and lung lymph nodes were collected from each dissected piglet, and RT-qPCR was used to measure the viral load in these organs. The animal study was approved by the Animal Ethics Committee of Sichuan Agricultural University (Approval Number: 20220261).

### 2.8 Statistical analysis

Statistical analysis was performed with GraphPad Prism 8.0.2 (GraphPad Software, San Diego, CA, United States). All data were subjected to one-way ANOVA followed by t-tests to determine statistical significance. A value of *p* of less than 0.05 was considered statistically significant.

## 3 Results

### 3.1 Virus isolation and identification

**Figure 1:**
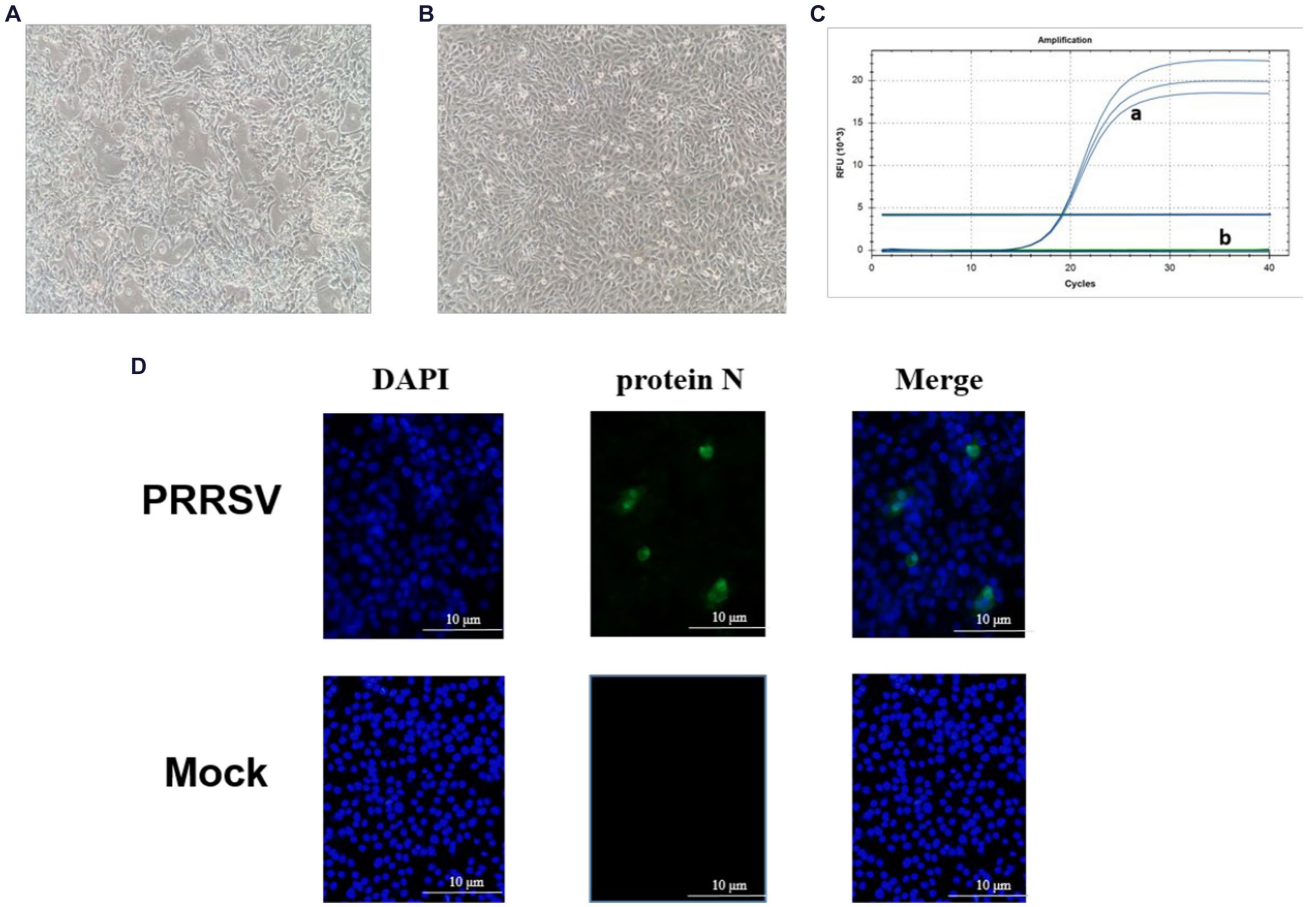
Isolation and Identification of the PRRSV isolate. **(A)** CPE diagram of Pams-163 infected with PRRSV isolate; **(B)** Blank control; **(C)** RT-qPCR results of the PRRSV isolate: (a) Amplification results of NADC30-like PRRSV (3 Replicates). (b) Amplification results for Highly Pathogenic-PRRSV, Classical PRRSV, NADC34-like PRRSV; **(D)** Results of indirect immunofluorescence assay.

PRRSV-positive specimens were inoculated onto monolayer Pams-163 and cultured in a 37°C CO_2_ incubator for 4–5 days. At 48 h post-inoculation with PRRSV, Pams-163 exhibited CPE, including cell shrinkage, irregular cell margins, increased refraction, and partial detachment (Figure 1A). In contrast, the control cells remained healthy and maintained distinct cell boundaries (Figure 1B). Pams-163 supernatant, which showed 80% CPE after six continuous passages, was subjected to RT-qPCR testing for PRRSV using the primers listed in Table 1. The isolated strain was identified as an NADC30-like strain (Figure 1C). PRRSV proliferation on Pams-163 was confirmed through immunofluorescence assay (IFA), which demonstrated green fluorescence surrounding the cell nuclei, while the negative control showed no fluorescence signal, indicating that the isolated PRRSV strain could proliferate on Pams-163 (Figure 1D).

### 3.2 Results of TCID_50_ determination

The CPE observed in each well of the 96-well plate was meticulously recorded. Subsequently, the TCID_50_ was calculated based on these observations using the Reed-Muench formula. As shown in Supplementary Table S3, the TCID_50_ of the PRRSV isolate was determined to be 10^–6.5^/0.1 mL.

### 3.3 Plotting of viral multi-step growth curves

**Figure 2:**
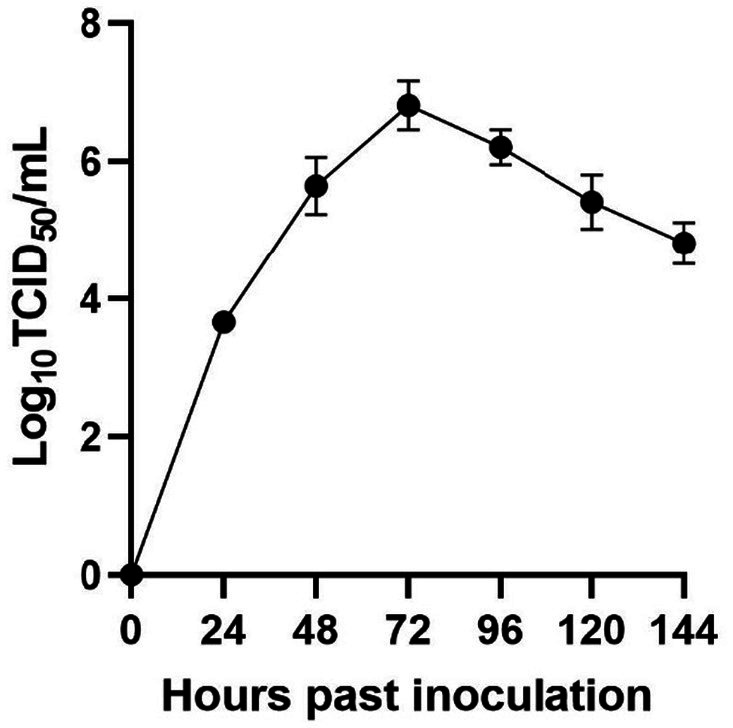
Viral multi-step growth curves.

By quantifying the TCID_50_ of the viral fluid at various time points, we constructed a correlation between the viral titers and time, thereby generating a multi-step growth curve for the virus (Figure 2). Our findings indicate that the titer of the isolate peaked at 72 h, reaching a value of 10^–6.74^/mL (MOI = 0.01), followed by a decreasing trend over time.

### 3.4 Phylogenetic and recombination analysis of PRRSV SCCD22

#### 3.4.1 Whole-genome sequencing and annotation of PRRSV SCCD22

**Figure 3:**
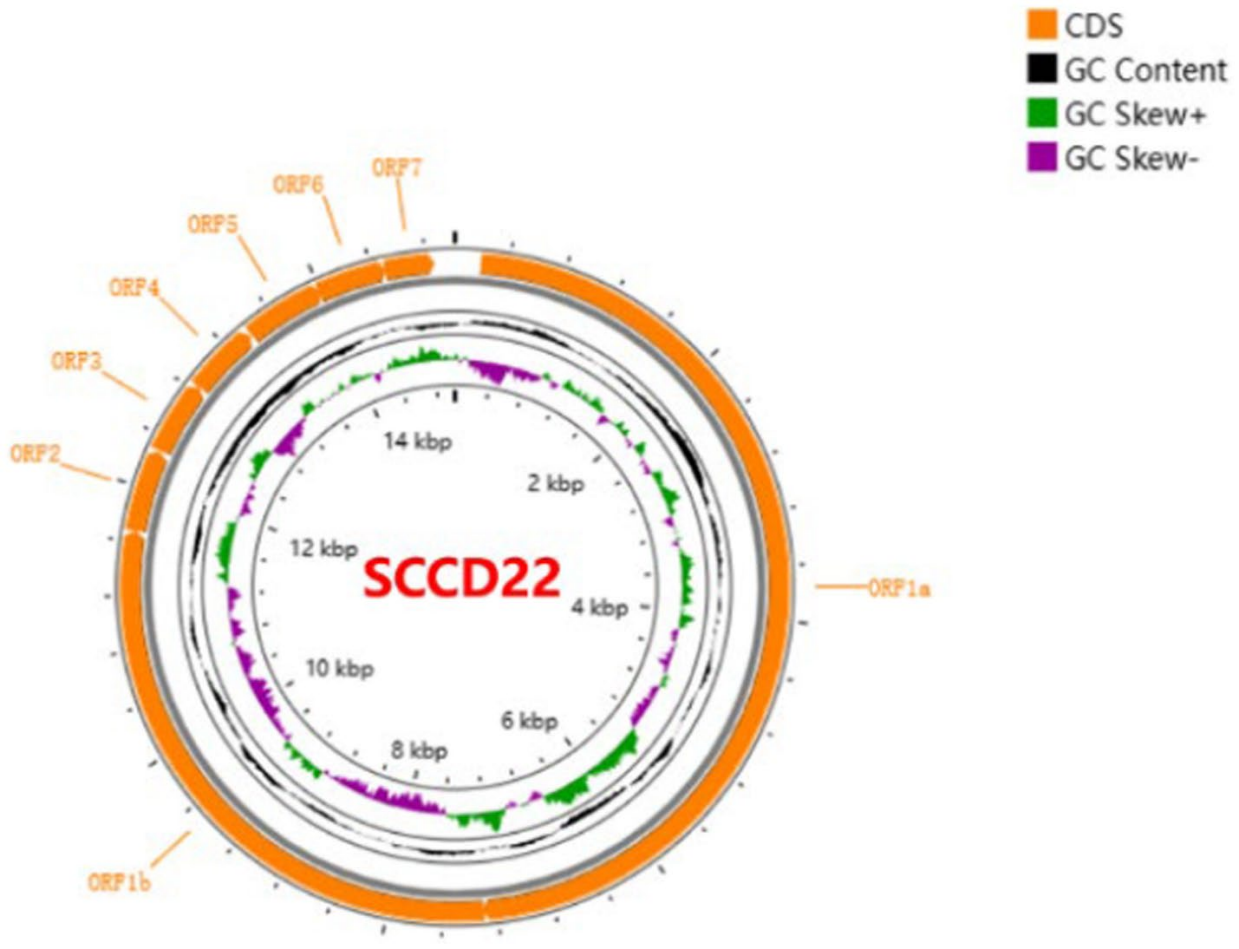
Whole genome sequencing and annotation of PRRSV SCCD22.

Utilizing Illumina sequencing technology, the whole genome of SCCD22 was sequenced, measuring 15,016 base pairs in length (GenBank accession number: OR670493.1). Visualization of the SCCD22 sequencing results using CGView revealed a GC content of 52.64% (Figure 3). The ORF1a and ORF1b regions constitute 75% of the entire PRRSV genome and are translated into two polyproteins, pp1a and pp1ab. The ORF2, ORF2b, ORF3, ORF4, ORF5a, ORF5, ORF6, and ORF7 regions encode the envelope proteins (E, GP2, GP3, GP4, GP5a, GP5, and M protein) and the nucleocapsid protein (N protein), respectively.

#### 3.4.2 Genetic evolution analysis of PRRSV SCCD22

To evaluate the genetic evolutionary relationships of PRRSV SCCD22 with reference strains from different lineages, we compared the nucleotide homology of SCCD22 with representative strains across various lineages (Table 2). The results indicated that SCCD22 shares a nucleotide identity ranging from 79.84 to 93.02% with strains CH-1a, JXA1, NADC30, NADC34, VR2332, and QYYZ, but only 58.60% with the LV strain, suggesting that SCCD22 belongs to PRRSV-2. Further comparative analysis revealed a close relationship between PRRSV SCCD22 and NADC30, with nucleotide and amino acid sequence identities in ORF1a, ORF1b, ORF3, ORF4, ORF5, ORF6, ORF7, and the 3’ UTR ranging between 81.70–97.30% and 84.38–98.85%, respectively.

**Table 2:** Sequence comparison results (in percentages) between PRRSV SCCD22 and other reference strains.

	CH-1a		JXA1		LV		NADC30		NADC34		VR2332		QYYZ	
	nt	aa	nt	aa	nt	aa	nt	aa	nt	aa	nt	aa	nt	aa
Complete genome	83.02	–	82.75	–	58.60	–	93.02	–	84.49	–	81.91	–	79.84	–
5’ UTR	94.24	–	95.79	–	52.91	–	93.19	–	92.63	–	92.11	–	92.15	–
ORF1a	78.71	77.83	78.98	78.94	53.21	45.78	91.11	89.33	79.88	80.07	78.81	78.63	74.23	74.34
ORF1b	75.51	82.77	74.40	82.67	54.42	58.30	81.70	84.38	76.91	83.12	75.67	82.50	73.55	81.95
ORF2	86.38	87.11	86.51	87.89	65.24	63.67	74.42	73.48	85.75	83.98	87.29	68.69	66.77	69.30
ORF3	84.05	82.28	83.79	81.89	63.17	56.39	94.38	94.88	85.74	84.65	83.92	84.65	82.48	83.64
ORF4	87.34	88.20	86.22	87.64	65.22	66.67	95.16	93.82	95.16	94.94	87.34	85.39	85.29	85.96
ORF5	86.73	85.50	85.74	85.50	59.22	50.99	92.21	92.50	87.56	90.00	85.74	85.50	83.91	83.50
ORF6	88.19	92.53	88.38	93.68	70.48	79.89	96.76	98.85	93.14	93.68	90.10	94.83	89.33	93.68
ORF7	91.40	92.68	90.32	91.06	60.86	57.25	95.43	98.37	95.70	97.56	92.47	95.12	87.63	91.87
3’ UTR	88.08	–	75.84	–	50.91	–	97.30	–	94.04	–	55.41	–	81.63	–

**Figure 4:**
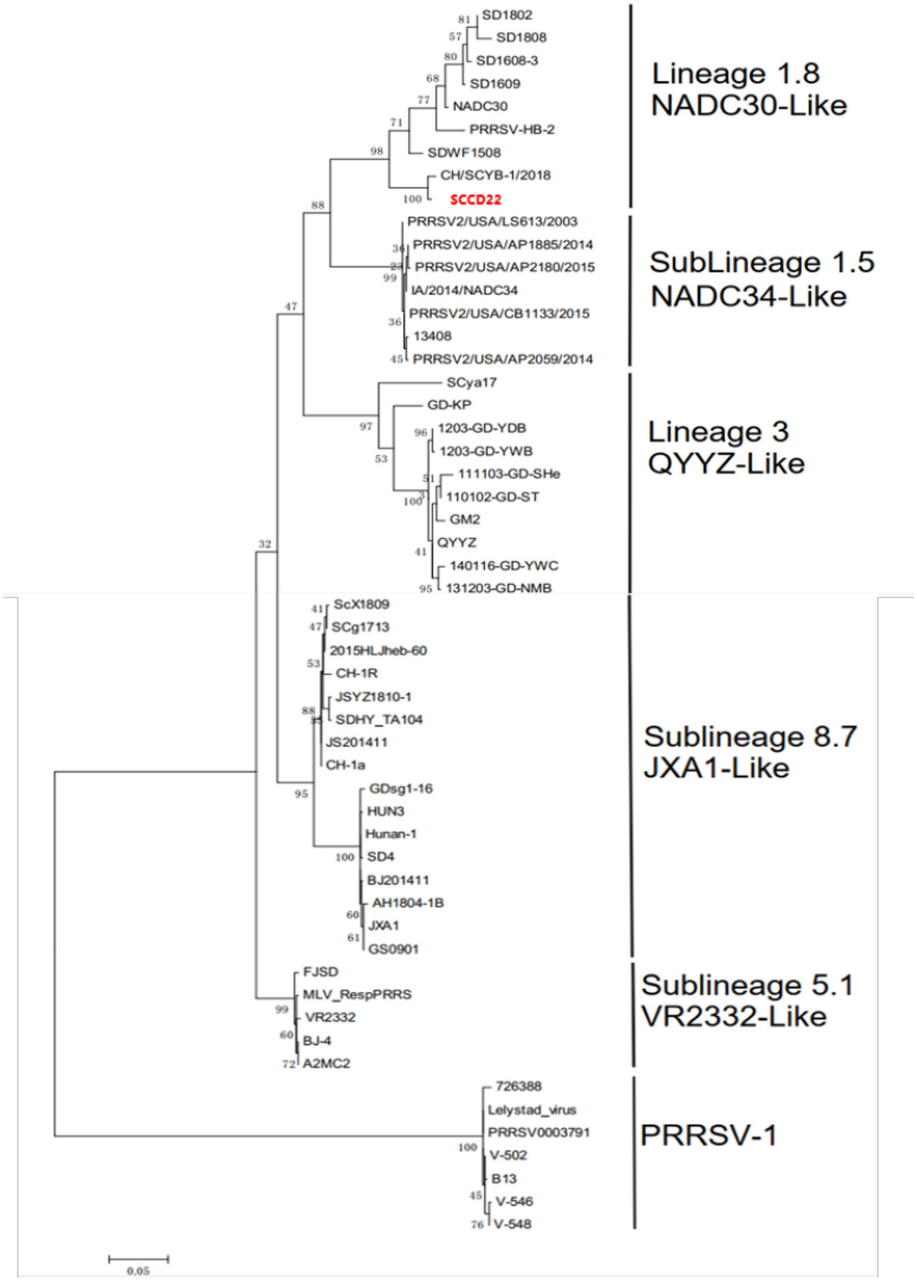
Phylogenetic tree based on PRRSV ORF5 gene.

A phylogenetic tree was constructed based on the ORF5 gene sequences (Figure 4), revealing that aside from PRRSV-2 strains, the remaining strains could be divided into four lineages based on the ORF5 gene. SCCD22 was classified within Lineage 1.8, showing the closest phylogenetic relationship with the CH/SCYB-1/2018 isolate from Yibin, China.

Comparison of the NSP2 amino acid sequence of SCCD22 with representative strains indicated that SCCD22, compared to the VR-2332 strain, exhibited three discontinuous deletions in NSP2 (111aa, 1aa, and 19aa) (Supplementary Figures S1A,B), aligning it with other NADC30-like strains.

**Figure 5:**
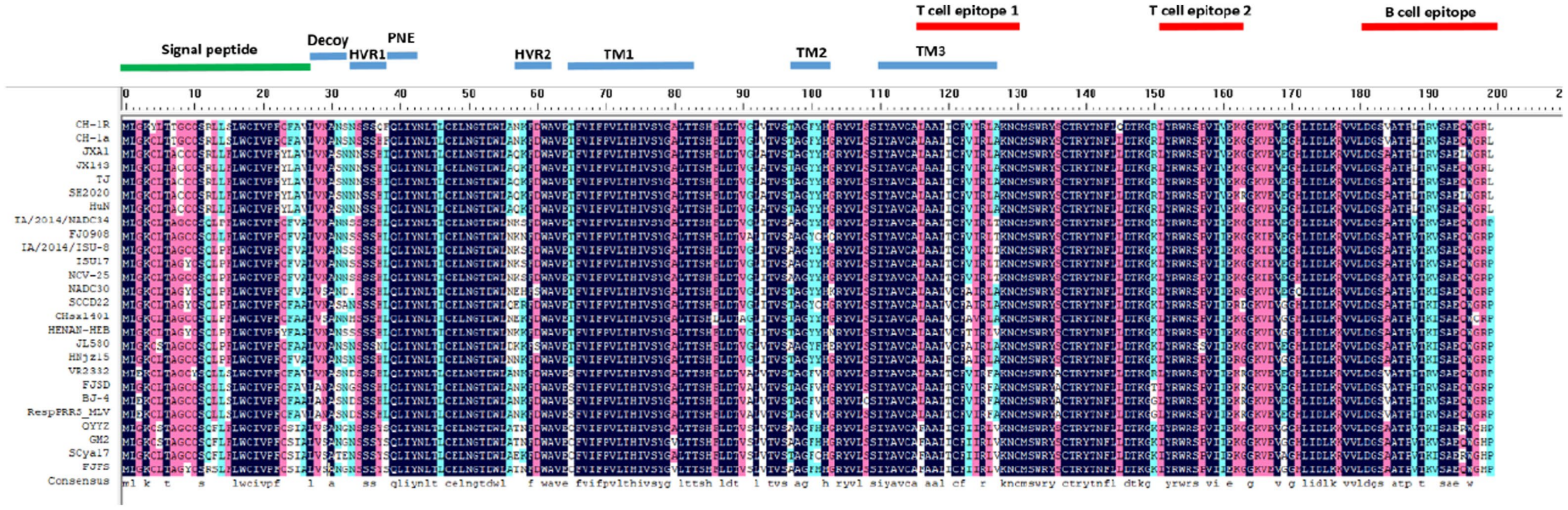
Comparison of ORF5 amino acid sequences among representative strains of PRRSV from different lineages.

Representative strains of different PRRSV lineages were selected to analyze ORF5 amino acid sequence variation. As shown in Figure 5, the results indicated that compared with the NADC30 strain, SCCD22 exhibits the greatest variation in the hypervariable region (HVR) 2, with a mutation in the 56-58aa NEH to QER. Additionally, a serine deletion was observed at position 33 in the hypervariable region 1 (HVR1).

#### 3.4.3 Recombination analysis of PRRSV SCCD22

**Figure 6:**
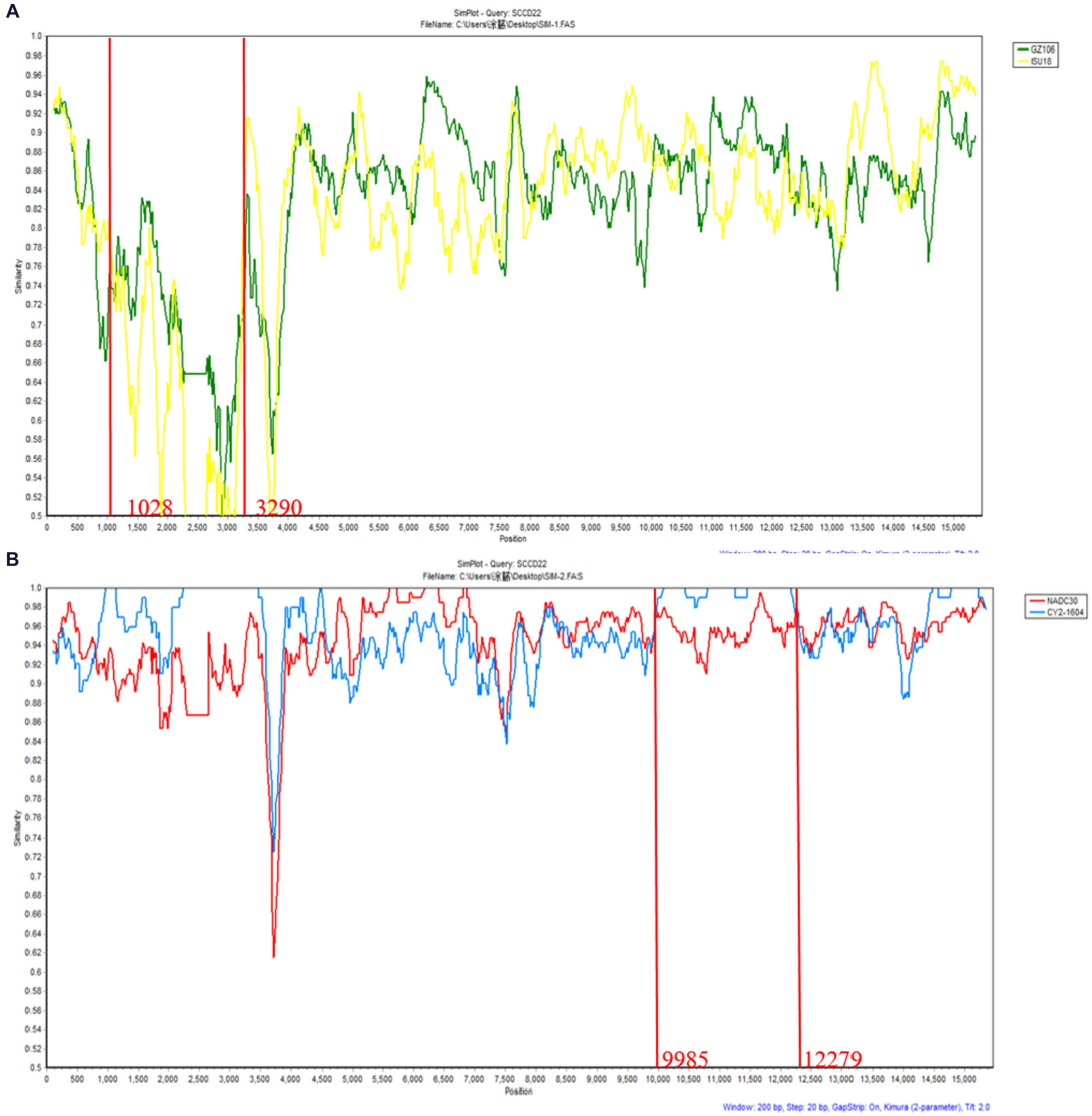
Genomic recombination analysis of SCCD22 using SimPlot. **(A)** GZ106 and ISU18were used as major and minor parents, respectively; **(B)** NADC30 and CY2-1604 were used as major and minor parents, respectively. Solid red lines indicate the recombination breakpoints; the locations are shown at the bottom.

Recombination events were predicted using RDPv5.1 and supported by at least three assays. Sequence similarity plotting was also performed using SimPlot 3.5.1 within a sliding window of 200 bp. The results (Table 3 and Figure 6) indicate two recombination events in the isolate SCCD22. In the first event, with GZ106 (sublineage 8.7) and ISU18 (sublineage 1.5) as the major and minor parents respectively, SCCD22 demonstrated a high sequence similarity to GZ106 in the 1,028–3,290 nt region. This region included a portion of the NSP2 coding area, where all seven detection methods of RDP5 identified significant *p* values. In the second event, with NADC30 (lineage 1.8) and CY2-1604 (lineage 1.8) as the major and minor parents, SCCD22 showed high similarity to CY2-1604 in the 9,985–12,279 nt region, which encompasses parts of the NSP10 to NSP12 coding areas, with all seven RDP5 detection methods again detecting *p* values.

**Table 3:** Summary of crossover events in SCCD22 identified by RDP5.

Recombined virus	Major parent	Minor parent	Region of recombination	*P* values for the seven assays in RDP5						
				RDP	GENECONV	Bootscan	Maxchi	Chimaera	Siscan	3seq
SCCD22	GZ106	ISU18	1,028–3,290 nt	4.197 × 10^−7^	1.888 × 10^−7^	3.130 × 10^−4^	8.222 × 10^−14^	1.461 × 10^−10^	9.976 × 10^−20^	3.290 × 10^−8^
	NADC 30	CY2-1604	9,985–12279 nt	2.026 × 10^−21^	7.431 × 10^−20^	4.308 × 10^−15^	1.748 × 10^−11^	5.262 × 10^−10^	1.509 × 10^−18^	6.367 × 10^−9^

### 3.5 Pathogenicity analysis of SCCD22

#### 3.5.1 Changes in clinical symptoms of piglets

**Figure 7:**
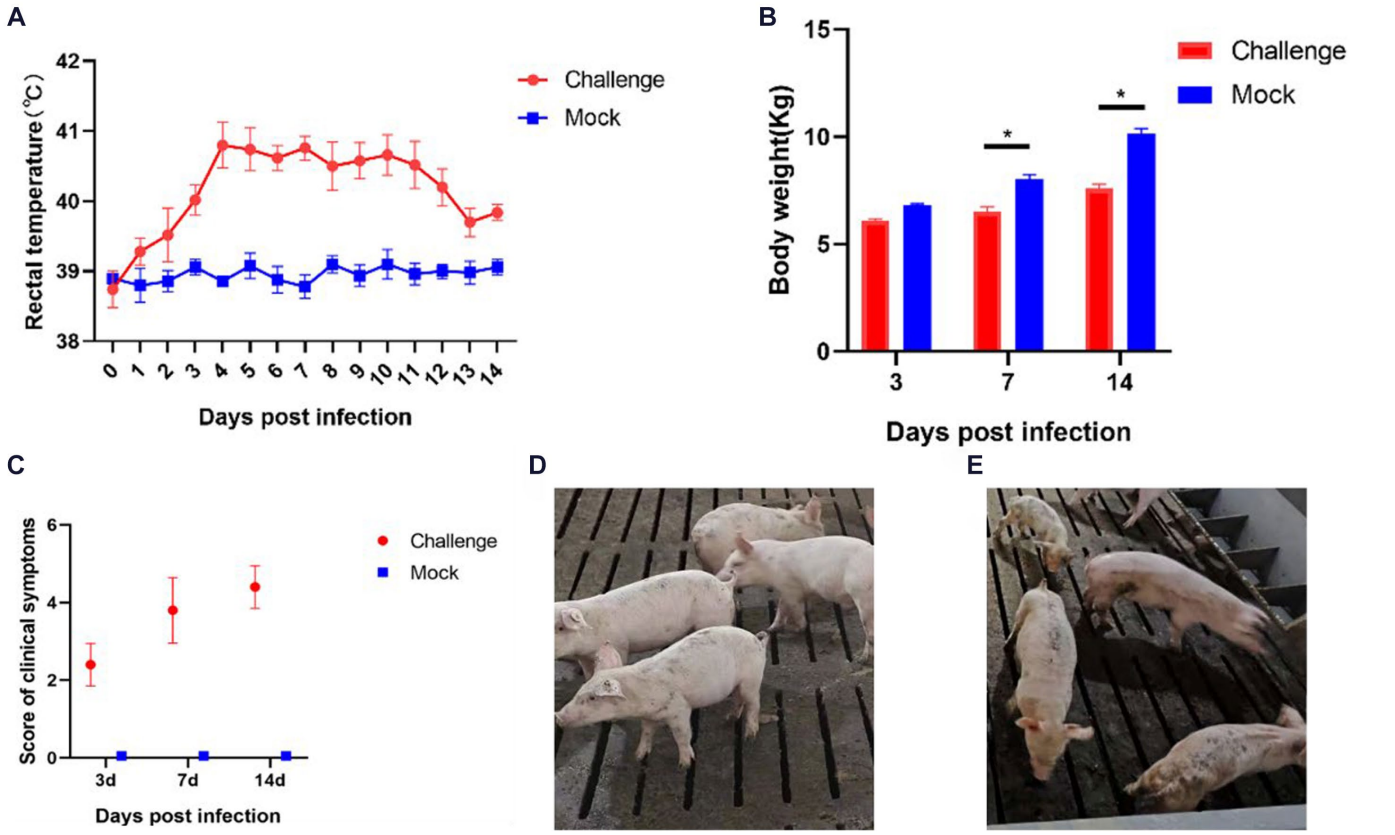
Clinical symptoms of the pathogenicity test in piglets. **(A)** Rectal temperature changes of piglets; **(B)** Body weight of piglets; **(C)** Clinical scores of piglets in each group during the whole experiment **(D)** Pigs in the Control group; **(E)** Pigs in the Challenge group. Significant differences are marked with asterisks, *p < 0.05.

After inoculation, a significant increase in body temperature was observed in the piglets, commencing from day 1 (Figure 7A). The temperature peaked at 41.2°C on the fourth day. Starting from day 4, all piglets in the inoculated group experienced sustained fever for 8 days, with temperatures consistently above 40°C. After day 12, there was a noted decline in the body temperature of the inoculated piglets. In contrast, the control group piglets exhibited no abnormal temperature fluctuations throughout the experiment.

Body weight measurements were conducted on days 3, 7, and 14 post-inoculation (Figure 7B). In the initial 3 days following inoculation, the body weights of the control and challenge groups showed no significant differences. However, from day 7, the body weight of the challenge group was significantly lower than that of the control group (*p* < 0.05). By day 14, the average weight of the control group piglets was 2.544 Kg heavier than the challenge group. These findings indicate that the isolate SCCD22 is capable of inhibiting weight gain in piglets.

After the challenge, we meticulously tracked and recorded the clinical symptoms in piglets from each group (Figure 7C). The challenge group piglets exhibited increasingly severe symptoms over time. In contrast, the control group piglets displayed no significant clinical symptoms (Figure 7D). From day 3, the challenge group piglets began showing decreased appetite and intermittent, mild coughing. On day 7, these piglets started clustering together, with a notable reduction in appetite, and some exhibited symptoms such as coughing, lethargy, and disheveled fur. Individual piglets showed distinct symptoms, including redness around the ears and abdominal breathing. On day 14, the piglets in the challenge group were characterized by rapid breathing, unkempt fur, and minimal food intake. Among them, one piglet displayed particularly severe symptoms, including emaciation, cessation of feeding, and respiratory distress (Figure 7E).

#### 3.5.2 Shedding of virus and changes in blood virus titers in piglets

**Figure 8:**
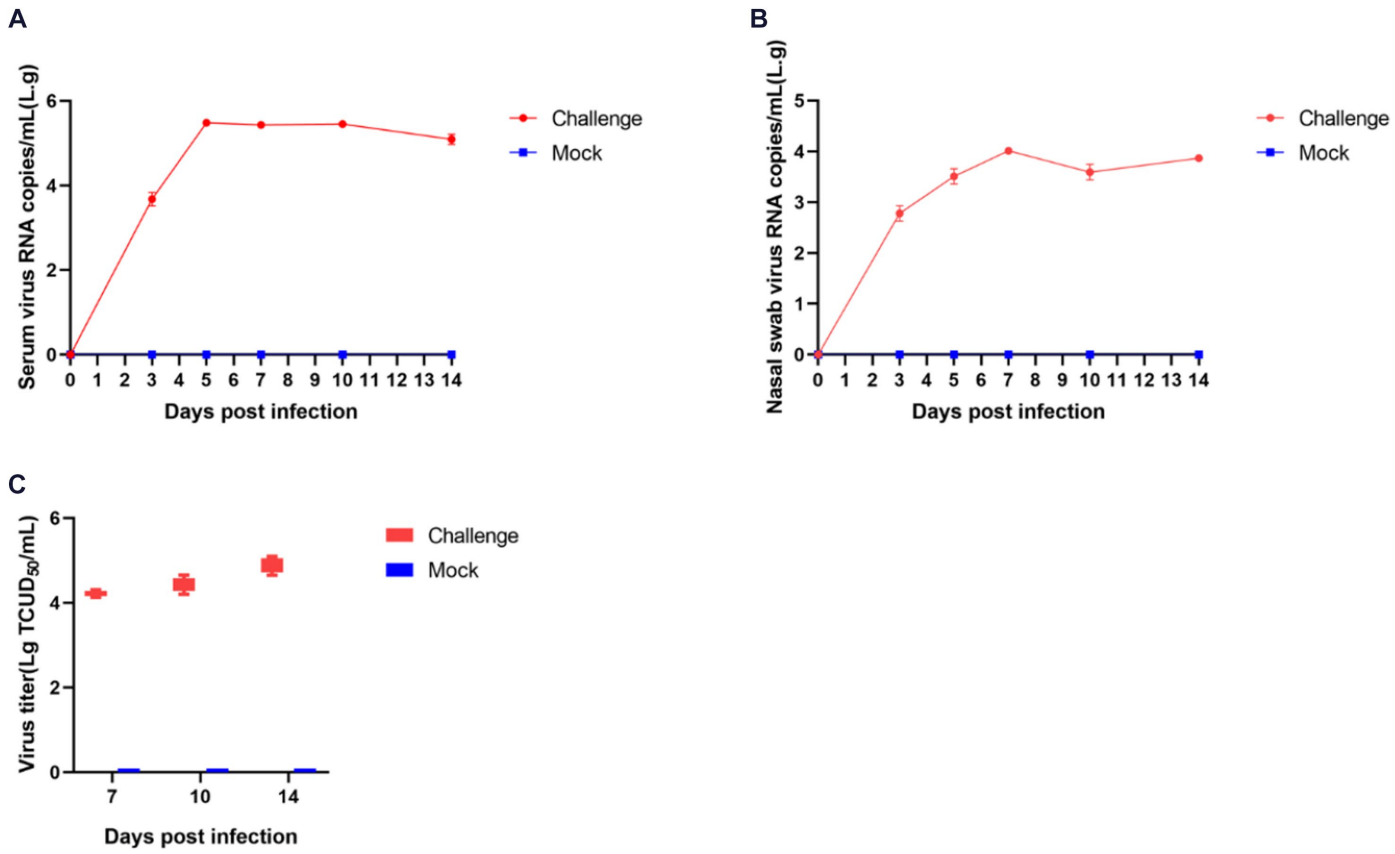
Shedding of virus and changes in blood virus titers in piglets after challenge. The amount of virus in serum **(A)** and nasal swabs **(B)**; Virus titers in the serum **(C)**.

The process of viral shedding in piglets and the changes in blood viral titers are shown in Figure 8. The serum viral load peaked on the fifth day post-infection at 3.65 × 10^5^ copies/mL, subsequently stabilizing above 8.69 × 10^4^ copies/mL until the end of the experiment (Figure 8A). Nasal swabs showed a peak viral concentration of 1.32 × 10^4^ copies/mL on the seventh day post-infection, followed by a decline (Figure 8B). The viral titers in serum on days 7, 10, and 14 post-infection were 10^–4.2^, 10^–4.4^, and 10^–4.8^ TCID_50_/mL, respectively, indicating a progressive intensification of viremia in piglets (Figure 8C).

#### 3.5.3 Assessment of viral loads in organs post-dissection and pathological observations

**Figure 9:**
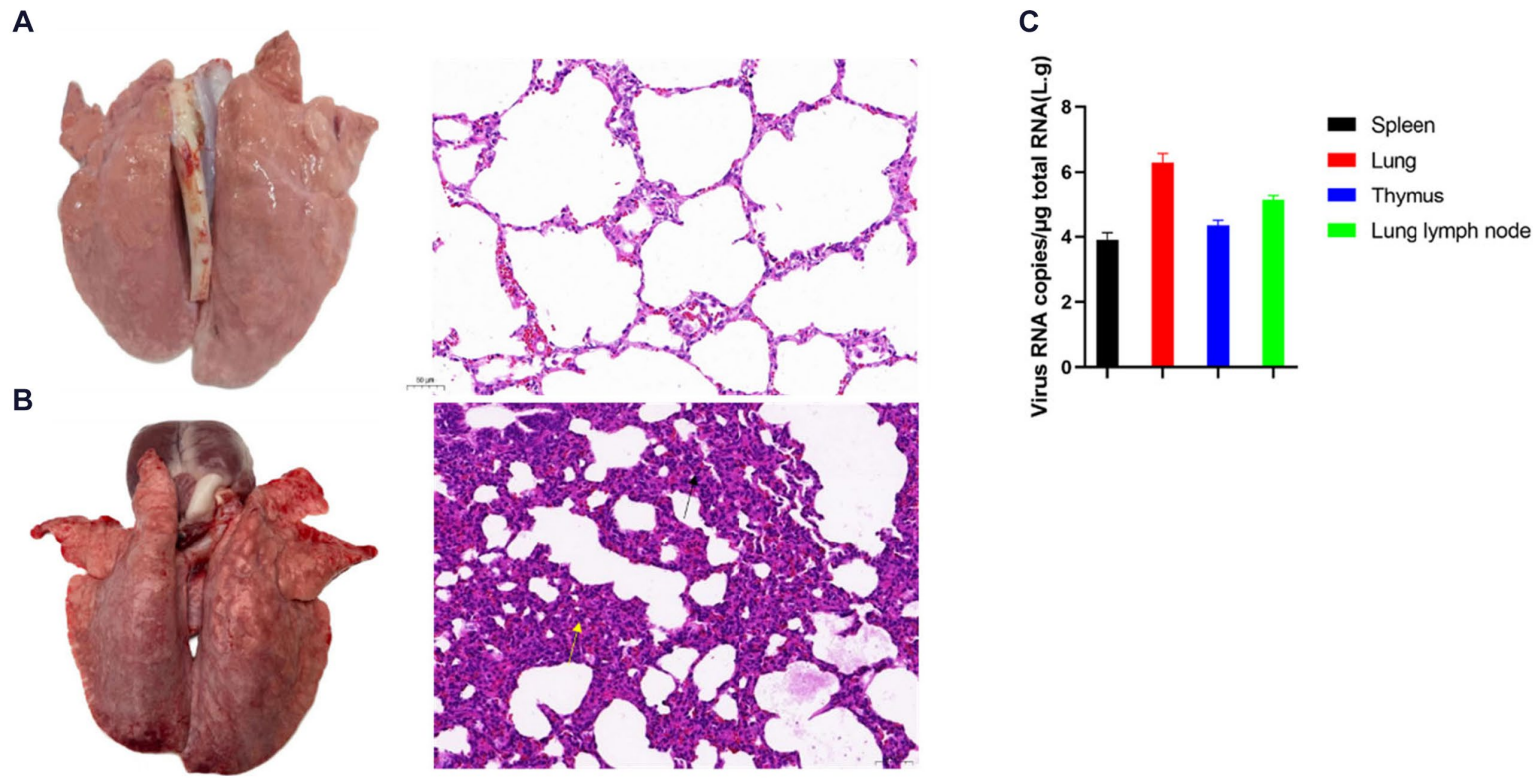
The pathological changes in the piglets’ lungs and the results of viral load testing after SCCD22 challenge. **(A)** Lung and histopathological sections from the control group; **(B)** Lung and histopathological sections from the challenged group; **(C)** Results of viral load in the organs of piglets from the challenge group.

In the control group, the piglets exhibited no visible lung abnormalities upon visual inspection (Figure 9A). Pathological section analysis revealed a normal lung structure with clear alveolar architecture. There were no signs of marked alveolar collapse or thickening of the alveolar walls, nor was there any congestion or expansion in the interstitial spaces or infiltration of inflammatory cells (Figure 9A).

In contrast, piglets in the challenge group predominantly displayed clear signs of pneumonia, characterized by either focal or diffuse distribution of lesions. The lesions varied in color from tan to dark purple, with some lung tissues undergoing consolidation and displaying a fish-scale pattern due to widened pulmonary interstitium (Figure 9B). Histopathological examination revealed an abnormal overall lung tissue structure. There was a significant increase in the width of the alveolar septa, resulting in substantial thickening, as indicated by yellow arrows. Additionally, conspicuous infiltration of inflammatory cells was observed, as indicated by black arrows (Figure 9B).

Viral loads in the spleen, lungs, thymus, and lung lymph nodes were quantified using RT-qPCR (Figure 9C). The highest viral concentration was observed in the lungs, followed by the lung lymph nodes and thymus, with the spleen registering the least viral presence. These results provide pivotal insights into the distribution and replication of the NADC30-like PRRSV recombinant strain in piglet organs, thereby advancing our understanding of its pathogenesis and transmission dynamics.

## 4 Discussion

Since its initial report in China in 2013, NADC30-like PRRSV has become widespread and shown an increasing annual clinical detection rate (Wang et al., 2017; Tu et al., 2023). Notable representative strains within China included HENAN-XINX (GenbankID: KF611905) and JL580 (GenbankID: KR706343). These strains exhibit a genomic homology of 86.7–87.8% with the U.S. isolate MN184A from 2001 (GenbankID: DQ176019), and 92.8–95.4% with the U.S. isolate NADC30 from 2008. Moreover, when compared with other strains like CH1a, jb-4, JXA1, and QYYZ, their homology varied between 82.2 and 87.2%. Similar to the U.S. isolates MN184A and NADC30, the Chinese NADC30-like strains also demonstrated the same NSP2 deletion pattern, including a discontinuous deletion of 131 amino acids (Zhao et al., 2015; Zhou L. et al., 2015; Li et al., 2016). The recurrence of this specific deletion across different geographic isolates may suggests its significance in the viral biology and pathogenesis, offering insights into potential targets for vaccine development and disease management strategies.

Several studies have demonstrated that recombination plays a pivotal role in the pathogenicity and genetic diversity of NADC30-like PRRSV in China. Notably, the virulence of these recombinant strains tends to be intermediate compared to their parental strains (Yu et al., 2021). Recombinant mutations observed in China’s NADC30-like PRRSV strains have undermined the cross-protective efficacy of existing vaccines, thereby posing significant challenges to the control and prevention of PRRSV in the region. Over recent years, our team has focused on collecting a wide array of PRRSV clinical samples to investigate the prevalence of the virus. Previous studies have reported an annual increase in the prevalence of NADC30-like PRRSV, particularly in Sichuan Province, China (Tu et al., 2022, 2023; Jiang et al., 2023). In a notable development, we isolated a mutant PRRSV strain from a clinical sample collected in 2022. This strain was named SCCD22 and contained recombinant fragments. Full genome sequencing revealed that SCCD22 is 15,016 bp in length, with a 93.02% genomic identity to NADC30. An evolutionary tree constructed based on ORF5 was used to classify SCCD22 into lineage 1.8 (NADC30-like PRRSV). This discovery adds a new dimension to our understanding of the viral evolution and spread, highlighting the need for continued surveillance and research in this field.

The NSP2 is one of the most variable regions in the PRRSV genome and is frequently targeted for monitoring genetic mutations in PRRS. Compared with VR2332, HP-PRRSV exhibits a discontinuous deletion of 30 amino acids (1aa + 29aa) in the NSP2 coding region (Tian et al., 2007), while NADC30-like PRRSV shows a discontinuous deletion of 131 amino acids (111aa + 1aa + 19aa) (Brockmeier et al., 2012), and NADC34-like has a continuous deletion of 100 amino acids (Bao and Li, 2021). In our analysis of the NSP2 amino acid sequence of the SCCD22 strain, we observed three discontinuous deletions (111aa, 1aa, and 19aa) compared with VR2332. This deletion pattern aligns with that of the NADC30-like PRRSV. This pattern merits further investigation as a marker for PRRSV surveillance and as a target in recombinant strain vaccine design. Additionally, the analysis of the amino acid sequence derived from the ORF5 gene showed significant variations in SCCD22 compared with the NADC30 strain, with the most substantial variation in HVR2, where amino acids 56–58 NEH were mutated to QER; and a Serine was deleted at position 33 in HVR1. These mutations and deletions highlight the ongoing and complex variability of PRRSV strains. This continuous genetic evolution presents a persistent challenge to the development and efficacy of control measures in the swine industry, particularly in China. Our study identified novel amino acid deletions and mutations in the SCCD22 strain, which have not been previously reported in the literature. These genetic alterations are potentially crucial for understanding the dynamic evolution of the virus’s pathogenicity. Furthermore, they could provide invaluable insights for the development of more effective vaccines and diagnostic methods, enhancing our ability to combat this evolving pathogenic threat.

Relative to other prevalent strains of PRRSV in China, the lineage 1.8, also known as NADC30-like PRRSV, demonstrates a heightened propensity for recombination and diverse pathogenicity. A review analyzing the pathogenicity of recombinant NADC30-like PRRSV prevalent in China indicated that the virulence of these domestic NADC30-like PRRSV is closely linked to their recombinant regions and the inherent virulence of the parental strains contributing to these segments (Yu et al., 2021). This study identified two notable recombination events in the SCCD22 isolate, specifically at 1028–3290 nt in the NSP2 and 9,985–12,279 nt in the NSP10 ~ 12. Concurrently, SCCD22 shows high similarity to GZ106 (sublineage 8.7) in the 1,028–3,290 nt, and to CY2-1604 (lineage 1.8) in the 9,985–12,279 nt, suggesting that the isolate SCCD22 may be a recombinant derivative of domestic NADC30-like (lineage 1.8) and JXA1-like (sublineage 8.7) strains. This intermingling of genetic traits from different lineages underscores the dynamic and complex nature of PRRSV evolution in China and highlights the continuous need for vigilant monitoring and in-depth genomic analyses to effectively manage and control this virus. In addition, this underscores the necessity for broadened surveillance, encompassing multiple lineages, to prevent and manage recombinant strain emergence effectively. Our investigation led to the discovery of novel recombination events in the NADC30-like PRRSV strain that have been previously undocumented in the scientific literature. These unique recombination events are important for elucidating the evolution of viral pathogenicity.

We further investigated the pathogenicity of SCCD22 in piglets. Our findings indicated that piglets in the challenge group exhibited elevated body temperatures with prolonged fever, reduced appetite, and rough coat conditions. Despite these clinical manifestations, there was no mortality was observed in piglets throughout the duration of the challenge. However, it was evident that the SCCD22 had a significant impact on the overall growth performance and weight gain of the piglets. This observation suggests that, in the absence of secondary infections, SCCD22 may not be directly responsible for post-weaning mortality in piglets. This finding is particularly relevant for understanding the clinical impact of PRRSV strains. Necropsies and histopathological examinations revealed that infection with SCCD22 in piglets led to typical pulmonary pathological changes characteristic of PRRSV infection. These changes included discernible alterations in the lung tissues, reinforcing the understanding that the lungs are the primary target organ for PRRSV. Moreover, after challenge, the piglets’ serum and nasal swabs showed prolonged viral shedding, and there was a gradual exacerbation of viremia. These findings underscore the persistence and potential for transmission of SCCD22, providing valuable insights into the pathogenic nature of this particular PRRSV variant. NSP2, a key component associated with viral packaging, manifests in various subtypes on the viral envelope and plays a pivotal role in the fusion process during the packaging of the Porcine Reproductive and Respiratory Syndrome Virus (PRRSV) (Zhao et al., 2018). NSP10, functioning as a helicase in PRRSV, is crucial for the unwinding of double-stranded RNA (dsRNA) and RNA synthesis during the virus’s replication cycle, thus being indispensable for effective virus replication (Bautista et al., 2002). NSP11 exhibits RNA endoribonuclease activity and deubiquitinating functions (Nedialkova et al., 2009; Fang and Snijder, 2010; Wang et al., 2015), while NSP12 promotes viral replication (Wang et al., 2015; 2019; Bai et al., 2020). In the case of SCCD22, the identified recombination events involving NSP2 and NSP10-12 are likely to have significant implications for the packaging and replication processes of PRRSV. This understanding is a critical part of our ongoing research on the behavior and pathogenicity of the virus. Our future research will aim to conduct *in vivo*/vitro studies to determine how these recombination events affect the pathogenicity of NADC30-like PRRSV. Furthermore, by acknowledging the coexistence of multiple PRRSV lineages, our study focused on investigating the epidemiology of PRRSV. This includes closely monitoring of the viral potential for recombination and its pathogenicity in the aftermath of such genetic exchanges. These efforts are crucial for advancing our understanding of PRRSV and developing more effective strategies for its control and management. Additionally, our challenge experiment design faced certain limitations. In the control group, the use of RPMI 1640 medium to inoculate piglets was not the optimal approach. A suspension of uninfected Pams-163 cells would have been a more appropriate choice. However, the swine-feeding site is characterized by a lack of facilities suitable for cell culture and preservation. Moreover, the significant distance between our laboratory and the swine-feeding site introduced logistical complexity. This is particularly relevant because extended transportation can detrimentally affect the viability of Pams-163 cells. In our methodology, the RPMI 1640 medium was employed as the foundational medium for both the viral cultures and the Pams-163 cell cultures in the negative control group, aligned with our experimental design parameters. Given these considerations, and to maintain consistency and control within our experimental framework, we used the RPMI 1640 medium as a negative control.

In summary, we successfully isolated and reported a new strain of NADC30-like PRRSV, which we designated as SCCD22. Our research encompassed a thorough analysis of this strain, delving into its genetic evolution, conducting a detailed recombination analysis, and assessing its pathogenicity. The insights gained from this study lay a crucial theoretical foundation for future advancement in this field. These findings are important for guiding the development of vaccines that specifically target NADC30-like PRRSV strains. Our research contributes valuable knowledge for broader efforts to control and prevent PRRSV infection. First, our findings can aid in the refinement of diagnostic assays. By incorporating newly identified genetic markers, these assays can become more sensitive and specific for detecting NADC30-like strains, enabling early detection and more effective management of outbreaks. Second, the unique genetic characteristics of the SCCD22 strain provide markers that can be used in molecular epidemiological studies. This can help track the spread and evolution of the virus, informing public health decisions and strategies for disease management. Third, understanding the specific mutations and recombination patterns in SCCD22 can guide the development of antiviral drugs that are more effective against these specific genetic profiles.

## Data availability statement

The datasets presented in this study can be found in online repositories. The names of the repository/repositories and accession number(s) can be found in the article/Supplementary material.

## Ethics statement

The animal study was approved by Sichuan Agricultural University Institutional Animal Care and Use Committee. The study was conducted in accordance with the local legislation and institutional requirements.

## Author contributions

TT: Conceptualization, Writing – original draft, Writing – review & editing. YLi: Conceptualization, Writing – review & editing. GZ: Writing – review & editing, Methodology. CD: Formal analysis, Writing – review & editing. YZ: Formal analysis, Writing – review & editing. DJ: Software, Writing – review & editing. YLuo: Funding acquisition, Writing – review & editing. XY: Writing – review & editing. ZY: Writing – review & editing. MR: Writing – review & editing. YW: Conceptualization, Funding acquisition, Writing – review & editing.

## Footnotes

^1^ https://proksee.ca/

## References

[ref1] Adams M. J. Lefkowitz E. J. King A. M. Q. Harrach B. Harrison R. L. Knowles N. J.. (2016). Ratification vote on taxonomic proposals to the international committee on taxonomy of viruses (2016). Arch. Virol. 161, 2921–2949. doi: 10.1007/s00705-016-2977-6, PMID: 27424026 PMC7086986

[ref2] Bai Y. Li L. Shan T. Zhang Y. Chen X. Gao F.. (2020). Proteasomal degradation of nonstructural protein 12 by RNF114 suppresses porcine reproductive and respiratory syndrome virus replication. Vet. Microbiol. 246:108746. doi: 10.1016/j.vetmic.2020.108746, PMID: 32605740

[ref3] Bao H. Li X. (2021). Emergence and spread of NADC34-like PRRSV in China. Transbound. Emerg. Dis. 68, 3005–3008. doi: 10.1111/tbed.14316 34492162

[ref4] Bautista E. M. Faaberg K. S. Mickelson D. McGruder E. D. (2002). Functional properties of the predicted helicase of porcine reproductive and respiratory syndrome virus. Virology 298, 258–270. doi: 10.1006/viro.2002.1495, PMID: 12127789 PMC7130902

[ref5] Brockmeier S. L. Loving C. L. Vorwald A. C. Kehrli M. E. Jr. Baker R. B. Nicholson T. L.. (2012). Genomic sequence and virulence comparison of four type 2 porcine reproductive and respiratory syndrome virus strains. Virus Res. 169, 212–221. doi: 10.1016/j.virusres.2012.07.030, PMID: 23073232

[ref6] Chai Z. (2009). Establishment of a differential method of highly pathogenic strain and classical strain of PRRSV by SYBR green-I real-time PCR assay. Chinese Acad. Agric. Sci. Master Dissertation. Available at: https://kns.cnki.net/kcms2/article/abstract?v=l-44aStnccA0rYkiHg-T1494lCovwWk_gYWY2msMVR6-FpXns01hrukCZjIXuDvQlwP1rbC8W7QxcBxjetD_7otM7GElyZnGsB7n_xe-ngSeBGFq-qPs7VYPa_OvAXPPdHEArMtu-5pBhkHzbjtlQw==&uniplatform=NZKPT&language=CHS

[ref7] Chang H. Zheng J. Qiu Y. Chen C. Li Q. Wu Q.. (2023). Isolation, identification, and pathogenicity of a NADC30-like porcine reproductive and respiratory disorder syndrome virus strain affecting sow production. Front. Vet. Sci. 10:1207189. doi: 10.3389/fvets.2023.1207189 37483283 PMC10360194

[ref8] Chen N. Ye M. Li S. Huang Y. Zhou R. Yu X.. (2018). Emergence of a novel highly pathogenic recombinant virus from three lineages of porcine reproductive and respiratory syndrome virus 2 in China 2017. Transbound. Emerg. Dis. 65, 1775–1785. doi: 10.1111/tbed.12952, PMID: 29992742

[ref1002] Chen N. Ye M. Huang Y. Li S. Xiao Y. Li X.. (2019). Identification of Two Porcine Reproductive and Respiratory Syndrome Virus Variants Sharing High Genomic Homology but with Distinct Virulence. Viruses. 11:875. doi: 10.3390/v11090875, PMID: 31540541 PMC6783987

[ref9] Fang Y. Snijder E. J. (2010). The PRRSV replicase: exploring the multifunctionality of an intriguing set of nonstructural proteins. Virus Res. 154, 61–76. doi: 10.1016/j.virusres.2010.07.030, PMID: 20696193 PMC7114499

[ref10] Feng Z. Hongtao C. Jun Z. Lu C. Xinwei W. Hongying L.. (2014). Identification and molecular epidemiology of porcine reproductive and respiratorsyndrome virus prevailing in Henan province from 2012 to 2013. Chinese J. Vet. Sci. 34, 1398–1410. doi: 10.16303/j.cnki.1005-4545.2014.09.002

[ref11] Han G. Lei K. Xu H. He F. (2020). Genetic characterization of a novel recombinant PRRSV2 from lineage 8, 1 and 3 in China with significant variation in replication efficiency and cytopathic effects. Transbound. Emerg. Dis. 67, 1574–1584. doi: 10.1111/tbed.13491, PMID: 31975574

[ref12] Han G. Xu H. Wang K. He F. (2019). Emergence of two different recombinant PRRSV strains with low neutralizing antibody susceptibility in China. Sci. Rep. 9:2490. doi: 10.1038/s41598-019-39059-8, PMID: 30792441 PMC6385303

[ref13] Jiang D. Tu T. Zhou Y. Li Y. Luo Y. Yao X.. (2023). Epidemiological investigation and pathogenicity of porcine reproductive and respiratory syndrome virus in Sichuan, China. Front. Microbiol. 14:1241354. doi: 10.3389/fmicb.2023.1241354, PMID: 37779701 PMC10533931

[ref14] Kappes M. A. Faaberg K. S. (2015). PRRSV structure, replication and recombination: origin of phenotype and genotype diversity. Virology 479-480, 475–486. doi: 10.1016/j.virol.2015.02.012, PMID: 25759097 PMC7111637

[ref15] Li Y. Xu G. Du X. Xu L. Ma Z. Li Z.. (2021). Genomic characteristics and pathogenicity of a new recombinant strain of porcine reproductive and respiratory syndrome virus. Arch. Virol. 166, 389–402. doi: 10.1007/s00705-020-04917-8, PMID: 33385245

[ref16] Li C. Zhuang J. Wang J. Han L. Sun Z. Xiao Y.. (2016). Outbreak investigation of NADC30-like PRRSV in South-East China. Transbound. Emerg. Dis. 63, 474–479. doi: 10.1111/tbed.12530, PMID: 27292168

[ref17] Liu Y. Li J. Yang J. Zeng H. Guo L. Ren S.. (2018). Emergence of different recombinant porcine reproductive and respiratory syndrome viruses, China. Sci. Rep. 8:4118. doi: 10.1038/s41598-018-22494-4, PMID: 29515183 PMC5841431

[ref18] Liu J. Liu C. Xu Y. Yang Y. Li J. Dai A.. (2022). Molecular characteristics and pathogenicity of a novel recombinant porcine reproductive and respiratory syndrome virus strain from NADC30-, NADC34-, and JXA1-like strains that emerged in China. Microbiol. Spectr. 10:e0266722. doi: 10.1128/spectrum.02667-22, PMID: 36354339 PMC9769985

[ref19] Liu J. K. Zhou X. Zhai J. Q. Li B. Wei C. H. Dai A. L.. (2017). Emergence of a novel highly pathogenic porcine reproductive and respiratory syndrome virus in China. Transbound. Emerg. Dis. 64, 2059–2074. doi: 10.1111/tbed.12617 28198110

[ref20] Lunney J. K. Fang Y. Ladinig A. Chen N. Li Y. Rowland B.. (2016). Porcine reproductive and respiratory syndrome virus (PRRSV): pathogenesis and interaction with the immune system. Annu. Rev. Anim. Biosci. 4, 129–154. doi: 10.1146/annurev-animal-022114-111025 26646630

[ref21] Ma X. Wang P. Zhang R. Zhao Y. Wu Y. Luo C.. (2022). A NADC30-like PRRSV causes serious intestinal infections and tropism in piglets. Vet. Microbiol. 268:109397. doi: 10.1016/j.vetmic.2022.109397, PMID: 35364367

[ref22] Meng X. J. Paul P. S. Halbur P. G. Lum M. A. (1995). Phylogenetic analyses of the putative M (ORF 6) and N (ORF 7) genes of porcine reproductive and respiratory syndrome virus (PRRSV): implication for the existence of two genotypes of PRRSV in the U.S.A. and Europe. Arch. Virol. 140, 745–755. doi: 10.1007/BF01309962, PMID: 7794115 PMC7086766

[ref23] Nedialkova D. D. Ulferts R. van den Born E. Lauber C. Gorbalenya A. E. Ziebuhr J.. (2009). Biochemical characterization of arterivirus nonstructural protein 11 reveals the nidovirus-wide conservation of a replicative endoribonuclease. J. Virol. 83, 5671–5682. doi: 10.1128/JVI.00261-09, PMID: 19297500 PMC2681944

[ref24] Qiu W. Meng K. Liu Y. Zhang Y. Wang Z. Chen Z.. (2020). Simultaneous detection of classical PRRSV, highly pathogenic PRRSV and NADC30-like PRRSV by TaqMan probe real-time PCR. J. Virol. Methods 282:113774. doi: 10.1016/j.jviromet.2019.113774, PMID: 31726113

[ref25] Shi M. Lam T. T. Hon C. C. Hui R. K. Faaberg K. S. Wennblom T.. (2010a). Molecular epidemiology of PRRSV: a phylogenetic perspective. Virus Res. 154, 7–17. doi: 10.1016/j.virusres.2010.08.014, PMID: 20837072

[ref26] Shi M. Lam T. T. Hon C. C. Murtaugh M. P. Davies P. R. Hui R. K.. (2010b). Phylogeny-based evolutionary, demographical, and geographical dissection of north American type 2 porcine reproductive and respiratory syndrome viruses. J. Virol. 84, 8700–8711. doi: 10.1128/JVI.02551-09, PMID: 20554771 PMC2919017

[ref27] Sui X. Guo X. Jia H. Wang X. Lin W. Li M.. (2018). Genomic sequence and virulence of a novel NADC30-like porcine reproductive and respiratory syndrome virus isolate from the Hebei province of China. Microb. Pathog. 125, 349–360. doi: 10.1016/j.micpath.2018.08.048, PMID: 30149129

[ref28] Tian K. Yu X. Zhao T. Feng Y. Cao Z. Wang C.. (2007). Emergence of fatal PRRSV variants: unparalleled outbreaks of atypical PRRS in China and molecular dissection of the unique hallmark. PLoS One 2:e526. doi: 10.1371/journal.pone.0000526, PMID: 17565379 PMC1885284

[ref29] Tu T. Pang M. Jiang D. Zhou Y. Wu X. Yao X.. (2023). Development of a real-time TaqMan RT-PCR assay for the detection of NADC34-like porcine reproductive and respiratory syndrome virus. Vet. Sci. 10:279. doi: 10.3390/vetsci10040279, PMID: 37104434 PMC10141196

[ref30] Tu T. Wang Y. Liao C. Y. Zhang P. F. Xiang M. Y. Yang Z. X.. (2022). Isolation and bioinformatics analysis of the NADC30_Like CJS01 strain of the porcine reproductive and respiratory syndrome virus. Vet. Ital. 58, 47–55. doi: 10.12834/VetIt.2182.14564.1, PMID: 36398670

[ref31] Wang D. Fan J. Fang L. Luo R. Ouyang H. Ouyang C.. (2015). The nonstructural protein 11 of porcine reproductive and respiratory syndrome virus inhibits NF-κB signaling by means of its deubiquitinating activity. Mol. Immunol. 68, 357–366. doi: 10.1016/j.molimm.2015.08.011, PMID: 26342881 PMC7112538

[ref32] Wang T. Y. Fang Q. Q. Cong F. Liu Y. G. Wang H. M. Zhang H. L.. (2019). The Nsp12-coding region of type 2 PRRSV is required for viral subgenomic mRNA synthesis. Emerg. Microbes Infect. 8, 1501–1510. doi: 10.1080/22221751.2019.1679010, PMID: 31631782 PMC6818116

[ref33] Wang L. J. Xie W. Chen X. X. Qiao S. Zhao M. Gu Y.. (2017). Molecular epidemiology of porcine reproductive and respiratory syndrome virus in Central China since 2014: the prevalence of NADC30-like PRRSVs. Microb. Pathog. 109, 20–28. doi: 10.1016/j.micpath.2017.05.021, PMID: 28512020

[ref34] Wu Y. Peng O. Xu Q. Li Q. Li W. Lin L.. (2022). Characterization and pathogenicity of two novel PRRSVs recombined by NADC30-like and NADC34-like strains in China. Viruses 14:2174. doi: 10.3390/v14102174, PMID: 36298730 PMC9607012

[ref35] Yu Y. Zhang Q. Cao Z. Tang Y. D. Xia D. Wang G.. (2021). Recent advances in porcine reproductive and respiratory syndrome virus NADC30-like research in China: molecular characterization, pathogenicity, and control. Front. Microbiol. 12:791313. doi: 10.3389/fmicb.2021.791313, PMID: 35087492 PMC8787316

[ref36] Yun Z. Zhixiong L. Ru C. Changbao L. (1998). Molecular cloning and identification of the ORF7gene of Chinese isolates B13 of porcine reproductive and respiratory syndrome virus. Chinese J. Vet. Med. 24, 3–5.

[ref37] Zhang Q. Jiang P. Song Z. Lv L. Li L. Bai J. (2016). Pathogenicity and antigenicity of a novel NADC30-like strain of porcine reproductive and respiratory syndrome virus emerged in China. Vet. Microbiol. 197, 93–101. doi: 10.1016/j.vetmic.2016.11.010, PMID: 27938690

[ref38] Zhang H. Leng C. Ding Y. Zhai H. Li Z. Xiang L.. (2019). Characterization of newly emerged NADC30-like strains of porcine reproductive and respiratory syndrome virus in China. Arch. Virol. 164, 401–411. doi: 10.1007/s00705-018-4080-7, PMID: 30353281

[ref39] Zhao K. Ye C. Chang X. B. Jiang C. G. Wang S. J. Cai X. H.. (2015). Importation and recombination are responsible for the latest emergence of highly pathogenic porcine reproductive and respiratory syndrome virus in China. J. Virol. 89, 10712–10716. doi: 10.1128/JVI.01446-15, PMID: 26246582 PMC4580157

[ref40] Zhao J. Zhiwen X. Ling Z. (2018). Research advances in non-structural protein 2 of porcine reproductive and respiratory syndrome virus. Acta Agric. Zhejiangensis 30, 350–356. doi: 10.3969/j.issn.1004-1524.2018.02.23

[ref1003] Zhou F. Zhao J. Chen L. Chang H. Li Y. Liu H.. (2015). Complete genome sequence of a novel porcine reproductive and respiratory syndrome virus that emerged in china. Genome Announc. 3, e00702–e00715. doi: 10.1128/genomeA.00702-15, PMID: 26159524 PMC4498110

[ref41] Zhou L. Wang Z. Ding Y. Ge X. Guo X. Yang H. (2015). NADC30-like strain of porcine reproductive and respiratory syndrome virus, China. Emerg. Infect. Dis. 21, 2256–2257. doi: 10.3201/eid2112.150360, PMID: 26584305 PMC4672414

