# Corrigendum: Isolation, identification, recombination analysis and pathogenicity experiment of a PRRSV recombinant strain in Sichuan Province, China

**DOI:** 10.3389/fmicb.2024.1391132

**Published:** 2024-03-11

**Authors:** Teng Tu, Yanwei Li, Guidong Zhang, Chengchao Du, You Zhou, Dike Jiang, Yan Luo, Xueping Yao, Zexiao Yang, Meishen Ren, Yin Wang

**Affiliations:** ^1^Key Laboratory of Animal Disease and Human Health of Sichuan Province, Sichuan Agricultural University, Chengdu, China; ^2^College of Veterinary Medicine, Sichuan Agricultural University, Chengdu, China

**Keywords:** porcine reproductive and respiratory syndrome virus (PRRSV), genetic recombination, lineage 1.8, pathogenicity, preventing and controlling

In the published article, the reference for Zhou et al., 2015 was incorrectly written as Zhou, L., Wang, Z., Ding, Y., Ge, X., Guo, X., and Yang, H. (2015a). Complete genome sequence of a novel porcine reproductive and respiratory syndrome virus that emerged in china. *Genome Announc*. 3, e00702–e00715. doi: 10.1128/genomeA.00702-15. It should be Zhou, F., Zhao, J., Chen, L., Chang, H., Li, Y., Liu, H., et al. (2015). Complete genome sequence of a novel porcine reproductive and respiratory syndrome virus that emerged in China. *Genome Announc*. 3, e00702-e00715. doi: 10.1128/genomeA.00702-15.

The authors apologize for this error and state that this does not change the scientific conclusions of the article in any way. The original article has been updated.
