# Supplementary material for: Isolation, identification, recombination analysis and pathogenicity experiment of a PRRSV recombinant strain in Sichuan Province, China
Source: Front Microbiol. 2024 Feb 21;15:1362471. doi: 10.3389/fmicb.2024.1362471 (PMC10915093; doi:10.3389/fmicb.2024.1362471)
Supplement: Supplementary file 1 [file Data_Sheet_1.DOCX]

Table S1. Reverse transcription system

| Reagent | Volume | reaction conditions |
| --- | --- | --- |
| gDNA Removal Reaction | 10 μL | 37°C for 15 min, 85°C for 5 s, and 4°C ∞ |
| EVO M-MLV Rtase Enzyme Mix | 1 μL |  |
| Oligo dT(18T)Primer | 1 μL |  |
| Random 6 mers Primer | 1 μL |  |
| 5×RTase Reaction Buffer MixI | 4 μL |  |
| RNase Free dH_2_O | 3 μL |  |
| Total | 20 μL |  |

Table S2. Clinical symptom scoring criteria

|  | Clinical symptom | Standard for evaluation | Score | |
| --- | --- | --- | --- | --- |
|  | Body temperature | T≤39.9℃ | | 0 |
|  |  | 40.0℃≤T≤40.9℃ | | 1 |
|  |  | 41℃≤T | | 2 |
|  | Appetite | Normal | | 0 |
|  |  | Loss of appetite | | 1 |
| Gross clinical symptom score |  | Hunger strike | | 2 |
|  | State of mind | Normal | | 0 |
|  |  | Unconsciousness/coma | | 1 |
|  | Skin | Normal | | 0 |
|  |  | Cyanosis | | 1 |
| Respiratory symptom score | Respiratory symptoms | Breathes fast when nervous | | 2 |
|  |  | Shortness of breath at rest | | 3 |
|  |  | Shortness of breath and difficulty breathing at rest | | 4 |
|  |  | Severe shortness of breath, difficulty breathing | | 5 |
|  | Cough | Normal | | 0 |
|  |  | Cough | | 1 |
|  | Runny nose | Normal | | 0 |
|  |  | Runny nose | | 1 |
|  |  | Normal | | 0 |
|  |  | Shiver | | 1 |
| Neurological symptom score | Neurological symptoms | Ataxia | | 2 |
|  |  | Limbs stroke | | 3 |
|  |  | Paralysis | | 4 |

Table S3. PRRSV isolate TCID50 determination results

| Degree of dilution | Number of cell Wells | Number of holes with CPE | Number of holes without CPE | The cumulative number of CPE holes | Cumulative number of holes without CPE | Total number of cell Wells | Percentage of CPE (%) |
| --- | --- | --- | --- | --- | --- | --- | --- |
| -1 | 8 | 8 | 0 | 48 | 0 | 48 | 100 |
| -2 | 8 | 8 | 0 | 40 | 0 | 40 | 100 |
| -3 | 8 | 8 | 0 | 32 | 0 | 32 | 100 |
| -4 | 8 | 8 | 0 | 24 | 0 | 24 | 100 |
| -5 | 8 | 8 | 0 | 16 | 0 | 16 | 100 |
| -6 | 8 | 5 | 3 | 8 | 3 | 11 | 72.73 |
| -7 | 8 | 3 | 5 | 3 | 8 | 11 | 27.27 |
| -8 | 8 | 0 | 8 | 0 | 16 | 16 | 0 |
| -9 | 8 | 0 | 8 | 0 | 24 | 24 | 0 |


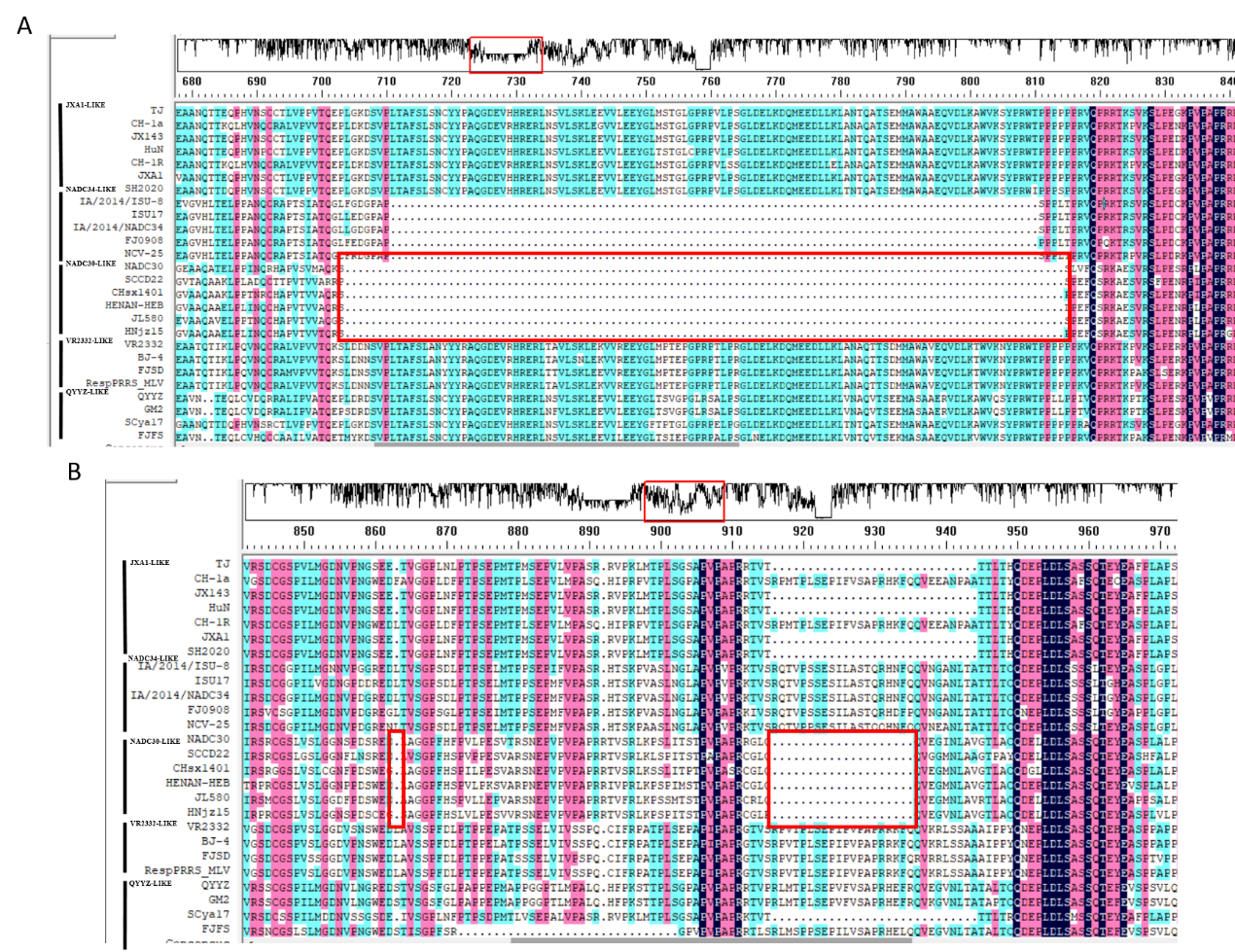


Figure S1 Amino acid sequence comparison of the NSP2 gene with representative strains from different lineages

(A) 680-840 aa; (B) 840-970aa
